# Oxygen‐Dependent Photoluminescence and Electrical Conductance of Zinc Tin Oxide (ZTO): A Modified Stern‐Volmer Description

**DOI:** 10.1002/cphc.202400984

**Published:** 2025-01-24

**Authors:** Linda Kothe, Josefin Klippstein, Marvin Kloß, Marc Wengenroth, Michael Poeplau, Stephan Ester, Michael Tiemann

**Affiliations:** ^1^ Faculty of Science – Department of Chemistry Paderborn University Warburger Str. 100 D-33098 Paderborn Germany; ^2^ Woehler Technik GmbH Woehler-Platz 1 D-33181 Bad Wuennenberg Germany

**Keywords:** Zinc tin oxide (ZTO), Gas sensor, Photoluminescence, Resistive sensor, Stern-Volmer model

## Abstract

Zinc tin oxide (ZTO) is investigated as a photoluminescent sensor for oxygen (O_2_); chemisorbed oxygen quenches the luminescence intensity. At the same time, ZTO is also studied as a resistive sensor; being an *n*‐type semiconductor, its electrical conductance decreases by adsorption of oxygen. Both phenomena can be exploited for quantitative O_2_ sensing. The respective sensor responses can be described by the same modified Stern‐Volmer model that distinguishes between accessible and non‐accessible luminescence centers or charge carriers, respectively. The impact of the temperature is studied in the range from room temperature up to 150 °C.

## Graphical abstract

A modified Stern‐Volmer model describes the impact of oxygen (O_2_) on both the photoluminescence intensity and the electrical conductivity of semiconducting zinc tin oxide (ZTO). The material is suitable as a O_2_ gas sensor by either optical or resistive principle.

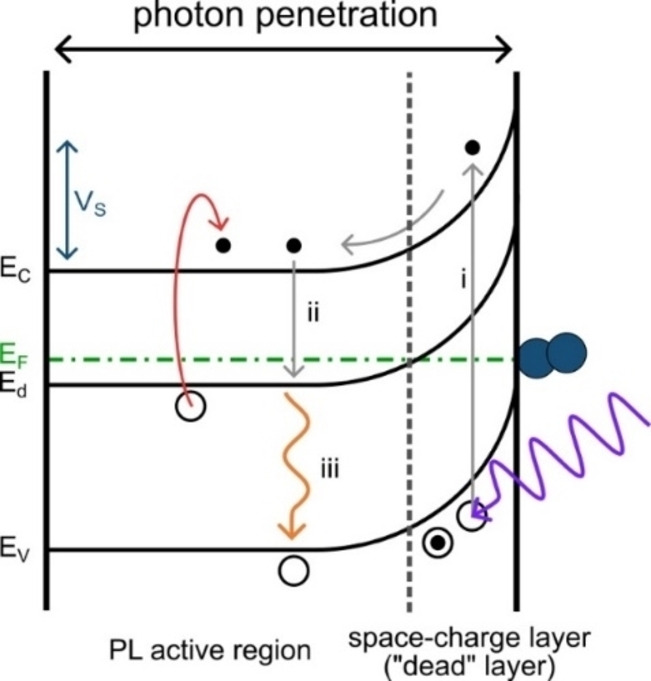

## Introduction

Photoluminescence‐based sensors have great potential for contactless gas detection.^[1]^ Most frequently, metal‐organic compounds are used. In recent years, however, purely inorganic materials were also investigated, including porous silicon,^[2]^ semiconducting metal oxides,[^3^, ^4^] and metal chalcogenides, including quantum dots.[^5^, ^6^] Oxygen (O_2_) detection is a major application of photoluminescence sensing. As oxygen is present in biological systems, safety and air quality, oxygen sensors are required to monitor vital or healing functions,[^7^, ^8^] detonation limits^[9]^ or air quality.^[10]^ Commercially available resistive O_2_ sensors based on metal oxides, *e. g*. zirconium dioxide (ZrO_2_), can cover a concentration range from 0.1 vol% to 100 vol% O_2_.^[11]^

To describe the gas‐dependent change of luminescence intensity and decay time of semiconductors, the ionosorption model, which is the basis of standard models in resistive semiconductor gas sensing, can be used. In case of *n*‐type semiconductors, oxygen (O_2_) binds to the surface, thereby immobilizing electrons from the conduction band, which reduces the overall conductance.^[12]^ This model can be extended to luminescence‐based sensors, where O_2_‐induced luminescence quenching is used for detection. Photoexcited electrons are trapped (immobilized) by adsorption of O_2_ at the surface and therefore cannot recombine radiatively or non‐radiatively with valence band holes or acceptor/donor states. With increasing O_2_ concentration, the thus‐induced electron depletion layer becomes thicker, and the surface barrier becomes higher. Hence, photoluminescence intensity and electrical conductance may be regarded as proportional.

At molecular level, O_2_‐induced quenching of photoluminescence is described by the Stern‐Volmer model.^[13]^ It has also been explored for quantum dots,[^6^, ^14^] but not to bulk solid‐state oxygen sensors, to the best of our knowledge. The Stern‐Volmer model distinguishes between static and dynamic quenching. In the static case, the luminescent center and a quencher molecule form a non‐radiative complex. This has an impact on the luminescence intensity but not on the decay time; the complex dissociates with increasing temperature. In case of dynamic quenching, the luminescent center and a quencher molecule collide, and the energy of the excited luminophore is transferred to the quencher. As the excited electron recombines non‐radiatively, the recombination rate is affected by the quencher molecule, although no chemical bond forms. With increasing temperature, more collisions occur, and the impact becomes more pronounced. The standard Stern‐Volmer Equation (1) applies to both mechanisms, where *I*_0_ and *I_Q_* are the fluorescence intensities in the absence and presence of the quencher, respectively, [*Q*] is the quencher concentration, and *K_SV_* is the Stern‐Volmer constant. The Stern‐Volmer equation is valid if all luminescent centers are equally accessible to the quencher molecules and either dynamic or static quenching occurs.

(1) $$\frac{I_{0}}{I_{Q}}=\;1+\;K_{SV}\;\cdot \;\left[Q\right]$$

Bulk materials may not be as accessible for the quencher as molecules, due to the coexistence of surface and bulk excitons. Therefore, we suggest the modified Stern‐Volmer Equation (2), which was first introduced by Lehrer^[15]^ for proteins in solution, as a suitable alternative that deepens the understanding of the sensing mechanism.

(2) $$\frac{I_{0}}{I_{0}-I_{Q}}=\;\frac{1}{f_{a}K_{a}\left[Q\right]}+\;\frac{1}{f_{a}}$$

The modified Stern‐Volmer equation contains a fraction *f_a_*, which provides quantitative information about the accessibility of the luminescent centers for the quencher.

Suitable metal oxides for photoluminescence‐based O_2_ sensing are, *e. g*., SnO_2_, TiO_2_, ZnO or lanthanoid‐doped ZrO_2_.[^3^, ^4^] Here we introduce zinc tin oxide (ZTO, Zn_2_SnO_4_) as another candidate. ZTO is an *n*‐type semiconductor which is chemically more stable than ZnO and transparent in the visible range (wide band gap of *ca*. 3.6 eV). It is interesting for a variety of potential applications, including as gas sensors, coatings, transistors, photocatalysts and in optoelectronics.[^16^, ^17^, ^18^] Therefore, ZTO is a suitable material for gas‐sensing studies based on both photoluminescence and electrical conductance.

In this work we show the O_2_‐depencence of the ZTO defective photoluminescence and investigate its quencher accessibility with the modified Stern‐Volmer equation between 40 °C and 150 °C.

## Results and Discussion

ZTO is used as an exemplary material to gain deeper understanding of photoluminescence and conduction O_2_ sensing. The synthesized ZTO is characterized in the Supporting Information (Figures S1–S5). Additional information about measurement setup and parameters are also available in the Supporting Information (Figures S6–S8).

### Sensing Mechanism

**Figure 1:**
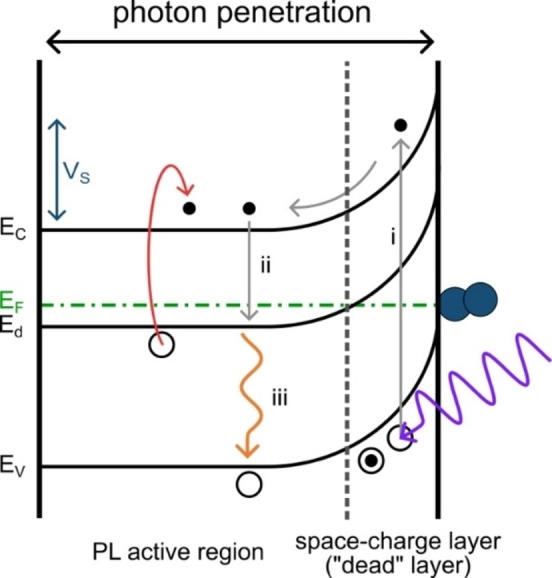
Schematic depiction of the energy levels of zinc‐tin oxide (ZTO). Excitation with UV radiation (violet), followed by non‐radiative transitions (gray) to the defect state, where electrons recombine radiatively (orange) with the valence band. Thermal activation (red) of electrons from the defect state to the conduction band.

For gas sensing, either by photoluminescence or by the resistive method, the surface‐near region of the semiconducting material is relevant (Debye length, photon penetration depth).[^19^, ^20^] Figure 1 shows a schematic of the band energies and defect state levels.

When the semiconductor is excited by electromagnetic radiation of higher energy than the band gap, electrons are elevated from the valence band to the conduction band (i). Since semiconductors show strong absorbance in the band gap energy domain, it can be assumed that the exciting radiation is completely absorbed and that all luminescent centers are excited. In case of ZTO, the band gap is between 3.6 eV and 3.7 eV. When ZTO is excited with 325 nm, the estimated photon penetration length, depending on the absorption coefficient (*e. g*., 0.625), is *ca*. 520 nm.^[21]^ Excited electrons from the conduction band reach a defect state by non‐radiative transition (ii). It was reported that photoluminescence in the blue‐green range is probably attributable to oxygen defects, whereas emission at longer wavelengths may be related to interaction between oxygen vacancies and interfacial tin or zinc vacancies.[^22^, ^23^] Hence, the photoluminescence in this study (orange, *ca*. 2.05 eV) is presumably based on a combination of interacting defect states. The excitation/emission spectrum and emission spectrum at λ_ex_=325 nm are shown in the Supporting Information section (Figure S4). From the defect state, electrons can recombine radiatively with valence band holes (iii).

The chemisorption of O_2_ results in an upward band‐bending, as ZTO is an *n*‐type semiconductor, and a decrease in Fermi energy. Photoexcited electrons are trapped by O_2_ at the surface and do not participate in recombination. Consequently, fewer photoelectrons are present, and the luminescence intensity decreases; an electron depletion layer is generated. It should be noted that different oxygen species must be expected to chemisorb to the ZTO surface, depending on the temperature. In case of SnO_2_ and ZnO the main adsorbing species between room temperature and *ca*. 80 °C is the neutral O_2_ molecule. In addition, the singly negatively charged O_2_^−^ molecular ion (superoxo ion) is present. This species implies a formal reduction of one O atom to the oxidation state ‐I and proves the chemical alteration of the metal oxide. At *ca*. 150 °C, the superoxo ion becomes more dominant than the uncharged molecule. The ratio of physisorbed (molecular) and chemisorbed (superoxo) O_2_ is temperature‐dependent, but only the chemisorbed species affects the photoluminescence and conductance. Above 200 °C, the dissociated O^−^ (peroxo) and O^2−^ (lattice‐like oxide) species are present.[^24^, ^25^, ^26^, ^27^] It is fair to assume a similar situation for ZTO. The supplemented surface charge due to chemisorption of O_2_^−^ and/or O^−^ is compensated by the space‐charge layer, as holes accumulate near the surface. With increasing temperature, the adsorption/desorption process of O_2_ is also accelerated.

The spatial separation of electrons and holes due to continuous excitation creates a space‐charge layer in the surface‐near regions of the metal oxide, where electrons move into the bulk and holes move to the surface. The presence of holes at the surface results in surface photovoltage and reduces band‐bending.^[28]^ The recombination rates are reduced by the near‐surface electric field. Due to the low PL intensity in this region, it is called “dead layer”.[^29^, ^30^] Therefore, the dead layer is a photophysical phenomenon. As band‐bending also affects the defect state, radiative recombination can only occur if the Fermi energy is larger than the defect state energy. The thickness of the space‐charge layer, which is roughly a synonym for the depletion layer, is defined as the depth where the Fermi and defect state energy are equal. The height of the depletion layer is called surface potential, *V_s_*, or Schottky barrier.

Beyond the space‐charge layer lies the photoluminescence‐active region, which is also affected by the surface potential and the thickness of the depletion layer. O_2_ saturation occurs if the Fermi energy is equal to the LUMO (lowest unoccupied molecular orbital) of O_2_ and no further band bending occurs. The LUMO of O_2_ is energetically lower than the Fermi energy of ZTO in the absence of O_2_. The decrease in Fermi energy due to O_2_ chemisorption is limited by the absolute position of the O_2_ LUMO. The energy difference between conduction band and Fermi energy stays intact. The absolute LUMO position of O_2_ does not change with O_2_ concentration, but the band bending and the absolute position of the Fermi energy, which are both limited by the absolute position of the O_2_ LUMO.

**Figure 2:**
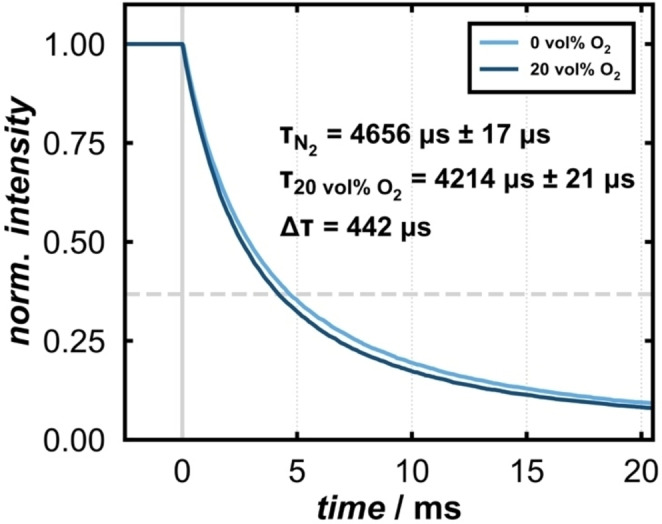
Life time of the orange emission (λ_em_=613 nm, with λ_ex_=325 nm) at room temperature in N_2_ (light blue) and 20 vol% O_2_ in N_2_ (dark blue).

In addition to the intensity the emission lifetime is also quenched in the presence of O_2_ (Figure 2). The emission spectrum (see Figure S4b) reveals that two emission bands overlap, resulting in an emission maximum at *ca*. 650 nm, when excited with 325 nm. The overlapping emission bands have their intensity maxima at 613 nm and 741 nm, respectively. Here we focus on the emission at 613 nm, while the emission at 741 nm will be part of future studies. To determine the lifetime, we evaluate the time that the system needs to decay to 1/*e* of the intensity. In a pure N_2_ atmosphere the lifetime is 4656 μs ± 17 μs, whereas with a content of 20 vol% O_2_, it is shorter by 442 μs (4214 μs ± 21 μs). The lifetimes under ambient conditions and at 741 nm are displayed in the Supporting Information, see Figure S5. The quenching of the lifetime supports the assumption, that the chemisorption of O_2_ introduces additional non‐radiative routes, which trap photoelectrons.

### Modified Stern‐Volmer Equation for Photoluminescence

**Figure 3:**
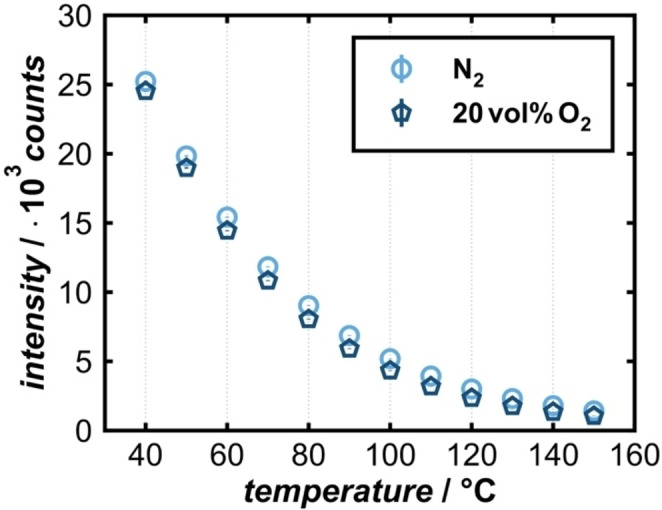
Temperature dependence of the intensity at 606 nm in N_2_ (light blue) and 20 vol% O_2_ in N_2_ (dark blue).

The difference in photoluminescence intensity in pure N_2_ and in a 20/80 vol% O_2_/N_2_ atmosphere is displayed in Figure 3. The emission intensity is higher in the absence of O_2_, which can be explained by the above‐described O_2_ quenching.

When a temperature change is applied, the intensity changes instantaneously. (Spikes in the intensity are artefacts caused by overdriving from the heating element.) With increasing temperature, more electrons from the defect state are thermally activated to the conduction band, which, in resistive gas sensing, causes an increase in electrical conductance. Concerning photoluminescence, however, non‐radiative recombination instances become more and more dominant with increasing temperature, resulting in temperature‐induced luminescence quenching. This is probably a Schön‐Klasens mechanism, since the thermal activation can also be measured via conductance and additional possibilities for non‐radiative recombinations are introduced by O_2_ chemisorption.[^31^, ^32^] The temperature quenching is likely affected by the chemisorption of O_2_ and therefore the respective species could have a different influence on temperature quenching. As previously described, the neutral molecular and the superoxo species are most likely present at the operating temperatures in this study. Therefore, the superoxo species might influence the temperature quenching.

**Figure 4:**
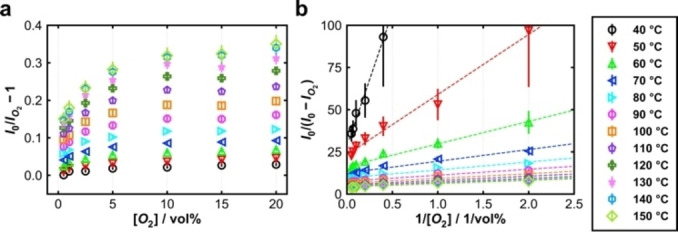
**(a)** Original Stern‐Volmer plot of photoluminescence between 40 °C and 150 °C. **(b)** Modified Stern‐Volmer plot of photoluminescence between 40 °C and 150 °C and the respective linear fits as dashed lines.

O_2_‐induced quenching was investigated by measuring the luminescence at variable O_2_ concentration (0 % … 20 vol% in N_2_), each at variable temperature. Data are plotted in Figure 4a as (*I*_0_/*I*_O2_)‐1 *vs*. O_2_ concentration, corresponding to the Stern‐Volmer Equation (1). (Measured intensities vs. time and vs. temperature are shown in the Supporting Information, Figure S8). Two findings are apparent from Figure 4a: (i) With increasing temperature, the slope of the graphs becomes steeper, which is a typical behavior of dynamic photoluminescence quenching. Oxygen chemically binds to the semiconductor's surface, analogous to the collision of the quencher molecule with the luminescent center in the description of molecular luminescence quenching. (ii) The relationship between (*I*_0_/*I*_O2_)‐1 and the O_2_ concentration is clearly not linear, *i. e*. Stern‐Volmer Equation (1) does not describe the behavior. The decrease in the slope of the graphs with increasing O_2_ concentrations indicates that not all luminescence centers interact with the quencher.^[13]^ This behavior is frequently observed for proteins or metal‐organic compounds with several luminescent centers.[^15^, ^33^] In addition, an inhomogeneous particle size distribution (see Figure S3a) may result in a variation of quencher accessibility.

For a more accurate description of the relationship between luminescence quenching and O_2_ concentration, we propose the extended Stern‐Volmer model in Equation (2). This equation is based on the assumption that there are two kinds of luminescent centers, only one of which interacts with the quencher molecules. The overall intensity is then the sum of both intensities. In Equation (2), I0_*,a*_ is the luminescence intensity (in the absence of the quencher) of the accessible centers that can interact with the quencher, with a Stern‐Volmer constant *K_a_*, and *I*_*0,b*_ is the luminescence intensity of the inaccessible centers that are unaffected by the quencher.

(2) $$I_{Q}=\;\frac{I_{0,a}}{1+\;K_{a}\left[Q\right]}+\;I_{0,b}$$

The fraction of accessible centers (irrespective of the quencher concentration) is defined as *f_a_* by Equation 3.

(3) $$f_{a}=\;\frac{I_{0,a}}{I_{0,a}+\;I_{0,b}}$$

Combining Equations (2) and (3) yields the modified Stern‐Volmer Equation (4) (see Supporting Information, Equations S2–S13.

(4) $$\frac{I_{0}}{I_{0}-I_{Q}}=\;\frac{1}{f_{a}K_{a}\left[Q\right]}+\;\frac{1}{f_{a}}$$

If *f_a_* equals 1, Equation (4) becomes the standard Stern‐Volmer Equation (1), as all centers are accessible.

**Figure 5:**
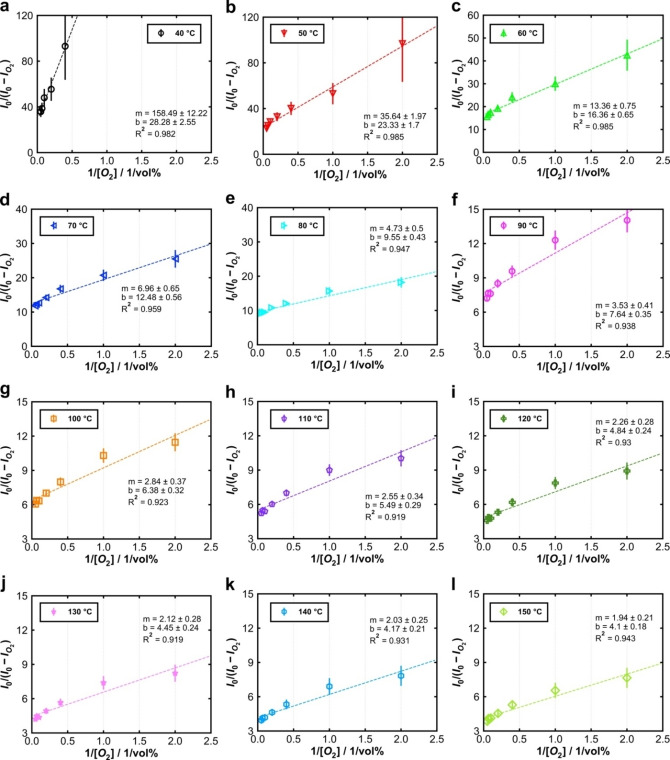
Modified Stern‐Volmer plot of photoluminescence and linear regression (dashed lines) at **(a)** 40 °C, **(b)** 50 °C, **(c)** 60 °C, **(d)** 70 °C, **(e)** 80 °C, **(f)** 90 °C, **(g)** 100 °C, **(h)** 110 °C, **(i)** 120 °C, **(j)** 130 °C, **(k)** 140 °C, and **(l)** 150 °C.

Figure 4b shows the plots of *I*_0_/(*I*_0_‐*I*_Q_) *vs*. [Q]^−1^, according to Equation (4), at variable temperature. All graphs show a near‐linear behavior for all temperatures; with increasing temperature, the slopes and intercepts decrease. Figure 5 shows the data in single plots (for clarity), including the results of linear regression (slope *m* and ordinate intercept *b*).

Between 40 °C and 60 °C the linearity (R^2^) is better than at higher temperatures. Since the modified Stern‐Volmer equation assumes two luminescent centers with only one of them interacting with the quencher, the deviation from linearity might indicate a third group of luminescent centers that also interact with the quencher but with a different Stern‐Volmer constant. Structural changes due to the increased measuring temperature seem negligible, as significant structural changes, such as opening of defect sites or formation of a porous phase, would be irreversible.

### Modified Stern‐Volmer Equation for Conductance

**Figure 6:**
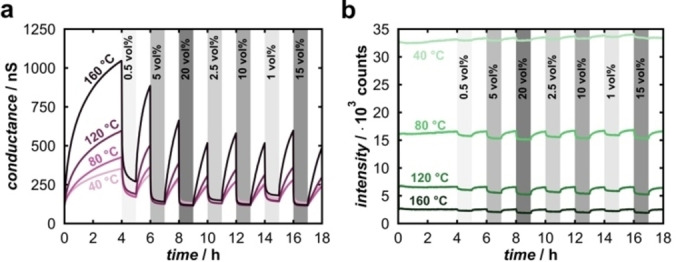
**(a)** Conductance and **(b)** photoluminescence measured simultaneously (40 °C, 80 °C, 120 °C and 160 °C) at different O_2_ concentrations.

As discussed above, a close relationship exists between O_2_‐induced luminescence quenching (usable for optical O_2_ sensing) and O_2_‐induced decrease in electrical conductance (usable for resistive sensing). Indeed, photoluminescence intensity and electrical conductance *G* turn out to be proportional to each other, as shown in Figure 6, which stands to reason considering that both are similarly affected by O_2_ adsorption.

The normalized measurements and concentration‐dependent values are displayed in the Supporting Information (Figures S10–S12). It needs to be stressed that the conductance was measured simultaneously with luminescence, *i. e*. under irradiation; without this optical activation, the conductance is altogether lower, as will be discussed below (see Supporting Information, Figure S13). The response is stronger for the conductance under all conditions, while photoluminescence shows faster response and a more stable signal baseline. In particular, the recovery of the conductance is vastly incomplete within the one‐hour periods between consecutive O_2_ supplies. The photoluminescence response to O_2_ increases with temperature, from 2 % at 40 °C to 25 % at 160 °C (for 20 % O_2_, as compared to pure N_2_ atmosphere). The lowest signal‐to‐noise ratio is observed at 120 °C.

**Figure 7:**
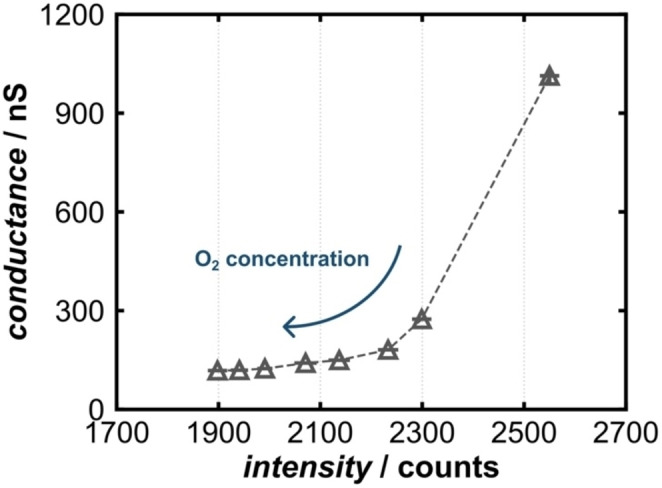
Conductance vs. photoluminescence at 160 °C. (The grey dashed line serves as a guide for the eye only).

At 160 °C, the O_2_ dependence for both conductance and photoluminescence is most pronounced. When plotted against each other, the proportionality of these two measurands is revealed (Figure 7).

The plot indicates two different proportionalities. Between 0 vol% and 2.5 vol% O_2_, a non‐linear relation exists, whereas between 2.5 vol% and 20 vol% O_2_, a linear proportionality manifests. This might indicate that at lower O_2_ concentrations mechanistic differences are more pronounced and need to be evaluated, but if saturation occurs, both measurands act roughly linear proportional with a slope of 0.143 nS/counts.

**Figure 8:**
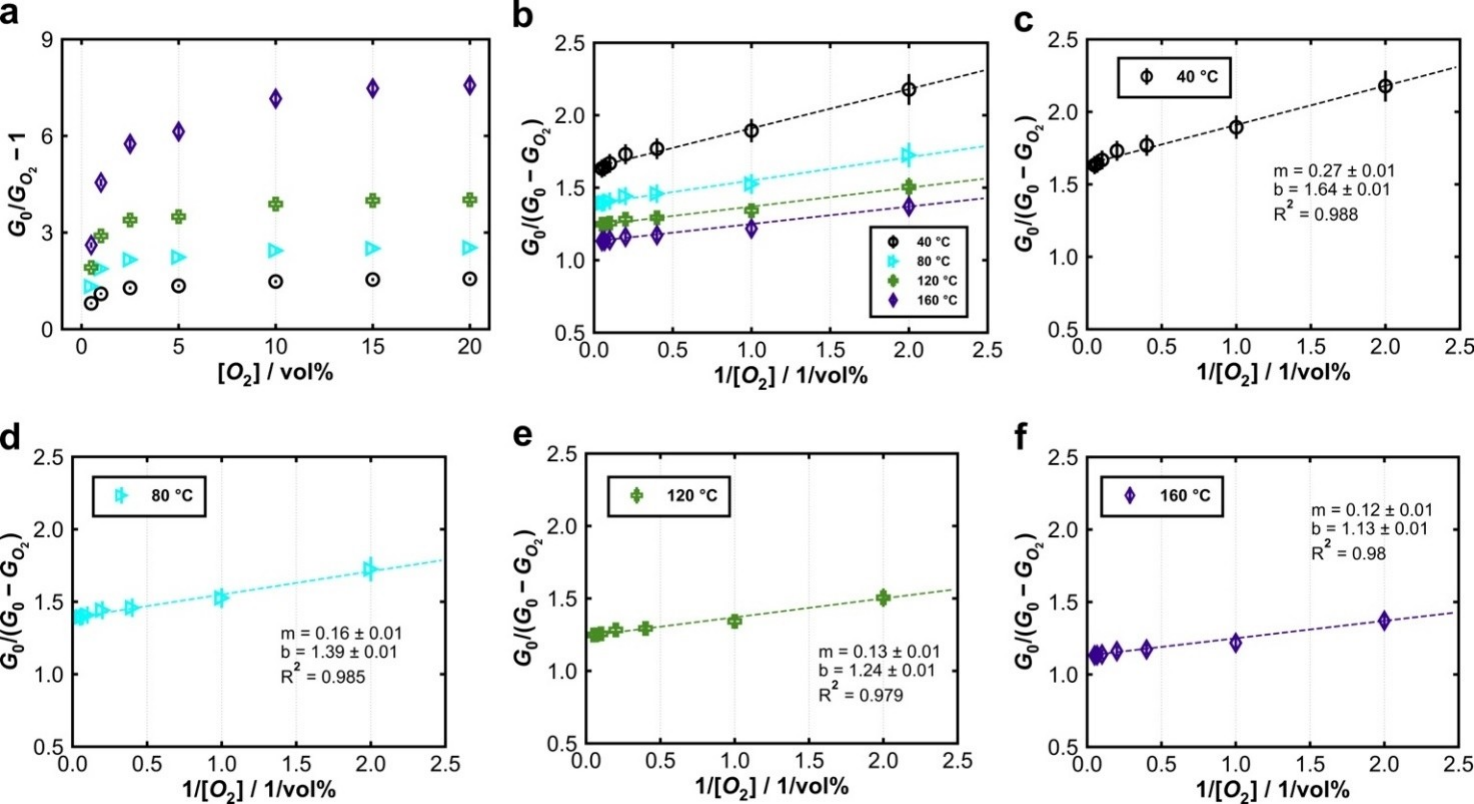
**(a)** Original Stern‐Volmer plot of conductance between 40 °C and 160 °C. Modified Stern‐Volmer plots of conductance and linear regression (dashed lines) between 40 °C and 160 °C **(b)** as well as single plots at **(c)** 40 °C, **(d)** 80 °C, **(e)** 120 °C, **(f)** 160 °C.

**Figure 9:**
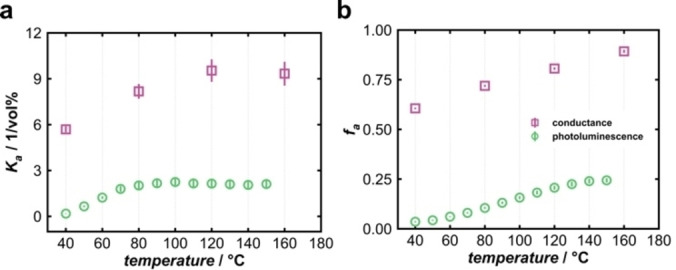
**(a)** Temperature‐dependent Stern‐Volmer constant *K_a_* and **(b)** accessible fraction *f_a_* for photoluminescence (green) and conductance (pink).

Applying the Stern‐Volmer Equation (1) to the electrical conductance *G* reveals the same behavior as previously observed for the luminescence intensity (Figure 8a). With increasing temperature, the slope of the graphs becomes steeper, and a downward curvature occurs. Hence, only a fraction of charge carriers seems to be accessible to O_2_. Again, when the data are plotted according to the modified Stern‐Volmer Equation (5), a near‐linear behavior is observed (Figure 8b), with the same tendencies as for the luminescence intensity. From the slopes and intercepts of the linear regressions to the data in Figure 4b and 7b, the Stern‐Volmer constant *K_a_* and the accessible fraction *f_a_* can be calculated from for each temperature (40 °C–160 °C). The results are shown in Figure 9 for both photoluminescence and conductance.

For luminescence, the Stern‐Volmer constant increases from *ca*. 0.18 vol%^−1^ (40 °C) to *ca*. 2.16 vol%^−1^ (90 °C) and stays approximately constant at higher temperature. The accessible fraction also increases with temperature. At 40 °C, less than 5 % (*f_a_* <0.05) of all luminescent centers are accessible to O_2_; for 150 °C, the value reaches *ca*. 25 % (*f_a_* =0.25). These results are consistent with the above‐mentioned ′dead layer’ model, especially for smaller temperatures, where the lowest degree of band‐bending occurs. Only few photo‐excited electrons are accessible to interact with O_2_ due to their spatial separation from the solid‐gas interface. With increasing temperature, more and more electrons are thermally activated from donor states below the conduction band (Figure 1), which is consistent with the observation of higher electrical conductance (see below). With a higher amount of charge carriers in the conduction band, more electrons can be trapped by O_2_. Thus, the surface potential increases, and the depletion layer becomes thicker.

Even though the Stern‐Volmer model can technically also be applied to the conductance data, the respective values of *f_a_* and *K_a_* are not physically analogous, though still proportional. The derivation of a corresponding model regarding the conductivity would be worthwhile and interesting. However, this would require additional studies based on adsorption models to gain a deeper understanding of the physical background of these parameters,[^34^, ^35^] which is beyond the scope of this study. Therefore, we compare the two distinct sets of *f_a_* and *K_a_* values at a qualitative level only. For electrical conductance, the Stern‐Volmer constant increases up to 120 °C, followed by a plateau. The *f_a_* values also become higher with temperature, which indicates that the charge carries might become more accessible to O_2_. For luminescence, both *K_a_* and *f_a_* reach a plateau at lower temperature than for conductance. This may indicate that recombination processes and, thus, the interaction with O_2_ are more affected by small temperature changes than in case of conductance.

We hypothesize that a major difference in accessibility between photoluminescence and conductance may be related to the binding energy of the excitons. The charge carriers, measured by conductance are mobile and mostly inhibited by the surface potential (Schottky barrier). However, in order for an exciton to release an electron to form a surface bond, the binding energy of the exciton must also be overcome. With increasing temperature, the exciton bonds become weaker, which may facilitate a higher degree of accessibility to O_2_.^[36]^ The separated excitons may act as additional luminescent centers that interact with O_2_, but with different kinetics as compared to intact excitons. It should also be stressed that, in case of photoluminescence, the Stern‐Volmer model indicates a dynamic quenching based on an energy transfer between the excited state and the quencher.

A pronounced influence of ultraviolet (UV) light illumination on both the electrical conductance and the response to oxygen (O₂) is observed. This is apparent from comparing the conductance measurements taken in the absence of illumination, as shown in the supporting information (Figure S13). This effect of photo‐activation is well‐established in the field of resistive gas sensing.[^37^, ^38^]

**Figure 10:**
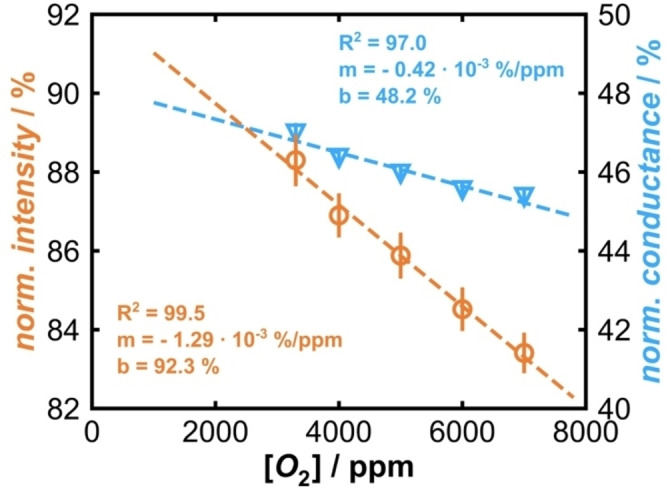
Normalized photoluminescence (orange) and conductance (light blue) measured simultaneously at 120 °C at different O_2_ concentrations (3300 ppm to 7000 ppm) both fitted by linear regression.

To estimate the lower O_2_ detection limit, photoluminescence and conductance were measured simultaneously at 120 °C with O_2_ concentrations between 3300 ppm and 7000 ppm (limits of the current gas mixing equipment, see Supporting Information, Figure S14). In previous studies we found that, at 120 °C, the signal‐to‐noise ratio due to O_2_ and temperature quenching is the highest.^[39]^ From the measurements, the normalized values at the different O_2_ concentrations were determined and plotted accordingly, Figure 10. For the measured concentration range a linear behavior is revealed.

Though the intercept of conductance is much lower compared to photoluminescence, the slope of the photoluminescence values is significantly higher. For a better comparison of the slopes, it was assumed for both measurands that the lower detection limit is the double value of the worst standard deviation for a reliable detection. For photoluminescence (1.32 %) results from the slope a detection limit of *ca*. 1000 ppm and for conductance (0.53 %) a limit of *ca*. 1250 ppm. This would indicate that photoluminescence might be more surface sensitive than conductance. The photoluminescence of SnO_2_ did not change significantly in the presence of O_2_, but the lower detection limit for the defect luminescence of TiO_2_ were at 25 ppm O_2_. For ZrO_2_ and ZnO no lower detection limits were determined so far, but O_2_ concentrations between 0.4 vol% and 2 vol% were usually detected reliably.[^3^, ^4^]

## Conclusions

Semiconducting zinc tin oxide (ZTO) was used as a sensor for oxygen (O_2_) by measuring both photoluminescence quenching (optical sensor) and electrical conductance (resistive sensing). An increase in temperature thermally quenches photoluminescence and enhances conductance by thermal activation. Luminescence intensity and electrical conductance are found to be proportional to each other, consistent with the fact that both phenomena are related to the degree of oxygen adsorption at the surface of ZTO. The responses to O_2_ (at elevated temperature) can be described by a modified Stern‐Volmer model that accounts for an accessible and for a non‐accessible fraction of luminescent centers and charge carriers, respectively. The accessible fraction of luminescent centers increased fivefold in a temperature range of 110 °C, which highlights the advantage of photoluminescence gas sensing at low operating temperature.

## Experimental

### Sensor Preparation

0.154 mol zinc(II)acetate (98 %; VWR) and 0.077 mol anhydrous tin(IV)chloride (99 %, VWR) were added to a solution of 150 mL absolute ethanol (≥99.5 %, VWR), 150 μL ethylene glycol (≥98 %, VWR) and 0.77 mol diethanolamine (synthesis quality, VWR). The synthesis mixture was refluxed for 2 h and then cooled to room temperature. The thus‐obtained clear solution (sol) was stored for one week. Part of the clear, colorless sol was heated to 550 °C with a heating rate of 300 °C/h and held for 4 h, followed by grinding. The residual was heated to 1100 °C with a heating rate of 300 °C/h and the temperature was kept for 6 h. Finally the product was ground. The substrates were cleaned alternately with acetone and ethanol, three times each, and dried at 80 °C. An aqueous dispersion of the product with a concentration of 0.04 mg/μL was prepared. At 80 °C 150 μL of the dispersion were drop coated twice on the interdigital structure of the substrate.

### Characterization

Scanning electron microscopy was performed with a Zeiss Neon 40. Powder X‐ray diffraction patterns were recorded with a Bruker D8 Advance diffractometer (Cu‐K_α,_ 0.02° steps, 3 s illumination time). The excitation‐emission spectra (2 nm steps) and lifetime measurements (“phosphorescence life time”, *λ_ex_=325 nm*, excitation bandwidth=5 nm, *λ_em_=613 nm* and 741 nm, emission bandwidth=20 nm, 50 ms chopping period) were recorded with a JASCO 8550 spectrofluorometer.

## Conflict of Interests

The authors declare no conflict of interest.

1

## Supporting information

As a service to our authors and readers, this journal provides supporting information supplied by the authors. Such materials are peer reviewed and may be re‐organized for online delivery, but are not copy‐edited or typeset. Technical support issues arising from supporting information (other than missing files) should be addressed to the authors.

Supporting Information

## Data Availability

The data that support the findings of this study are available from the corresponding author upon reasonable request.
