# Supplementary material for: Oxygen‐Dependent Photoluminescence and Electrical Conductance of Zinc Tin Oxide (ZTO): A Modified Stern‐Volmer Description
Source: Chemphyschem. 2025 Jan 24;26(7):e202400984. doi: 10.1002/cphc.202400984 (PMC11963967; doi:10.1002/cphc.202400984)
Supplement: Supplementary file 1 — Supporting Information [file CPHC-26-e202400984-s001.pdf]

ChemPhysChem

Supporting Information

Oxygen-Dependent Photoluminescence and Electrical Conductance of Zinc Tin Oxide (ZTO): A Modified Stern-Volmer Description

Linda Kothe, Josefin Klippstein, Marvin Kloß, Marc Wengenroth, Michael Poeplau, Stephan Ester, and Michael Tiemann*

Supporting Information

Characterization

This work focuses on the O_2-dependent photoluminescence and electrical conductance of zinc tin oxide (ZTO). The synthesis was inspired by Tsai et al.[40] As displayed in figure S1, Zn_2SnO_4 was obtained, with only low amounts of SnO_2.

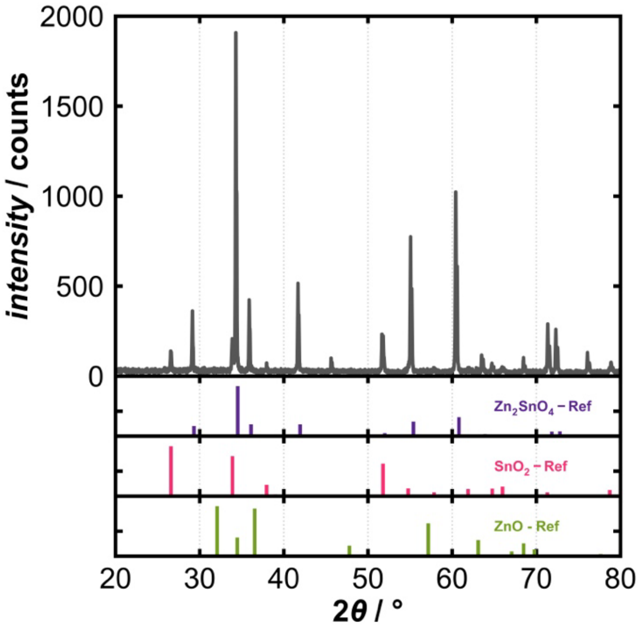

Figure S1. *x-ray diffraction pattern.*

An incomplete incorporation of SnO_2 is likely, since the formation of Zn_2SnO_4 is a solid-state reaction.[41] The most intense powder XRD reflection (311) at 34.30 ° was fitted with a linear combination of two gaussian functions, figure S2 and equation S1, to determine the center and full width at half maximum.

The (311) reflex of ZTO at 34.30 ° was fitted with a linear combination of two Gaussian functions, equation S1, as the Cu-$K_{\alpha,1}$ and Cu-$K_{\alpha,2}$ beam are not separated.

$$g(x) = A \frac{1}{\sigma\sqrt{2\pi}} exp\left(-\frac{1}{2}\left(\frac{x-\mu}{\sigma}\right)^2\right) \quad \text{(S1)}$$

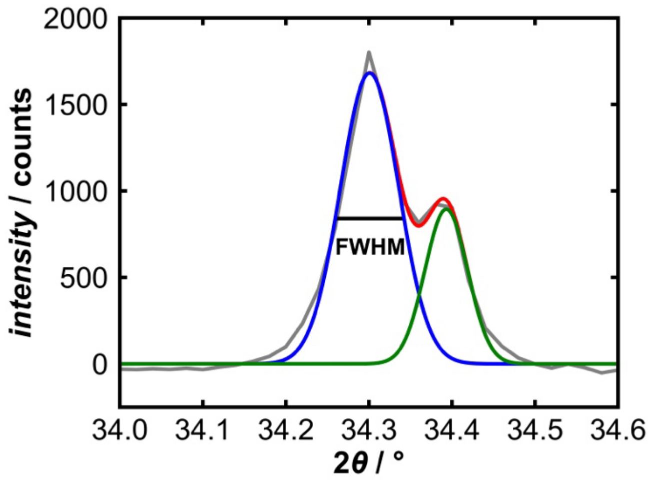

Figure S2. *magnification of x-ray diffraction (311) reflex at 34.30 °, fitted with a double gaussian function.*

Based on the Scherrer equation, the average crystallite size is 111.5 nm.[42]

This is consistent with scanning electron microscopy (SEM) images, figure S3a, as there are either smaller and bigger crystallites. Despite residual amounts of SnO_2, Zn and Sn are homogeneously distributed in the sample, figure S3b and c. Due to the different weights and resulting penetrations depths of X-rays, the topography of the particles is apparent in the energy dispersive X-ray spectroscopy (EDX) data.

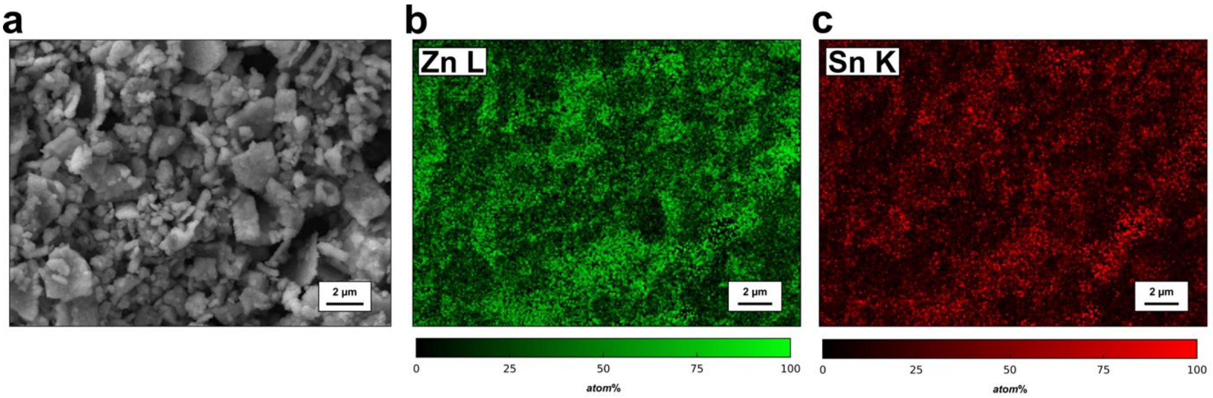

Figure S3. ***a*** *SEM image 5000 x magnification,* ***b*** *EDX mapping of Zn (green) and* ***c*** *Sn (red) in atom% 5000 x magnification.*

Excitation-Emission spectrum, figure S4a, and emission spectrum (λ_{ex} = 325 nm), figure S4b, at room temperature under ambient conditions of ZTO. The spectrum was fitted with a linear combination of two Gaussian functions. The two emission bands were also reported in the literature before.[22,23]

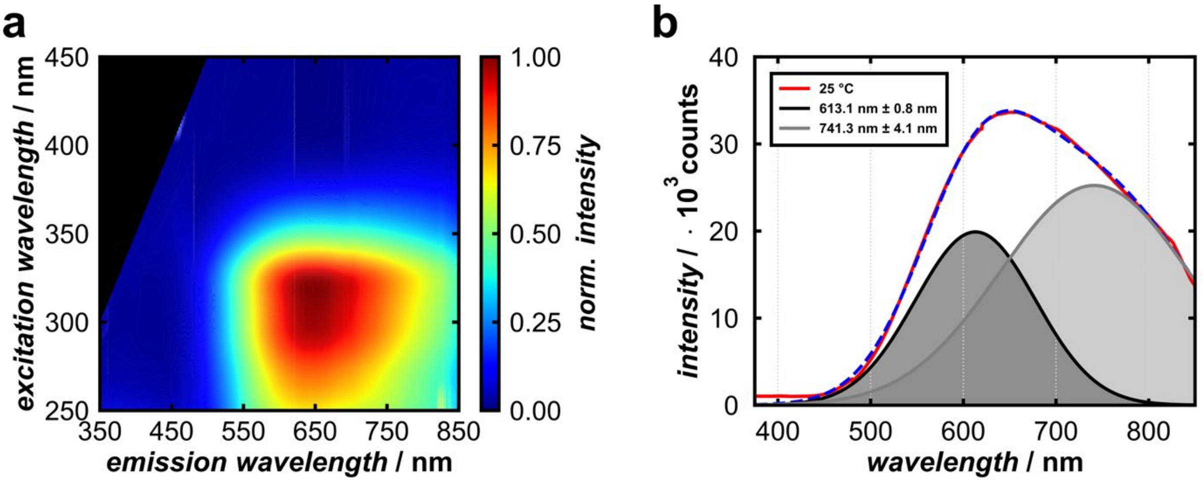

Figure S4. ***a*** *excitation-emission spectrum,* ***b*** *emission spectrum at room temperature fitted with a linear-combination of two Gaussian functions (λ_{ex} = 325 nm).*

The lifetime was determined at λ_{em} = 613 nm and 741 nm. Each measurement ("phosphorescence lifetime", λ_{ex} = 325 nm, excitation bandwidth = 5 nm, λ_{em} = 613 nm and 741 nm, emission bandwidth = 20 nm, 50 ms chopping period) was performed 10 times for each emission wavelength. One measurement is containing 100 measurements, which results in an average value of 1000 single measurements. As most solids don't show a single exponential decay behavior we evaluate the data in two different ways. The first method evaluates the time the intensity requires to reach a value of 1/e. A second method is based on the log-log scaling of the data, revealing an initial single exponential stage, followed by a finite stage, which can be fitted with a power law.[43] The slope of the power law might reveal additional information about different interactions, which can elongate the lifetime, e.g. exciton phonon interactions etc., but are not the focus of this work. We have chosen these methods to keep the determination of lifetimes as comparable as possible.

First the lifetimes at λ_{em} = 613 nm and 741 nm were determined under ambient (no active gas flow) conditions, figure S5a and b. Both evaluation methods reveal a longer lifetime of the 613 nm emission compared to 741 nm. In addition, the double-log scaling of the data indicate, that the emission at 613 nm might be more affected by additional interactions, as the elongation term is here much more pronounced. The effect of O_2 on the lifetime was then determined for both emission bands. The sample was kept in a pure N_2 or 20 vol% O_2 in N_2 atmosphere (300 ml/min) at room temperature for 1 h each before the measurements. The emission at 613 nm (Figure 2, Figure S5e) is quenched by O_2. In contrast the emission at 741 nm seems not to be quenched by O_2 (Figure S5c and S5d), which is subject for further studies.

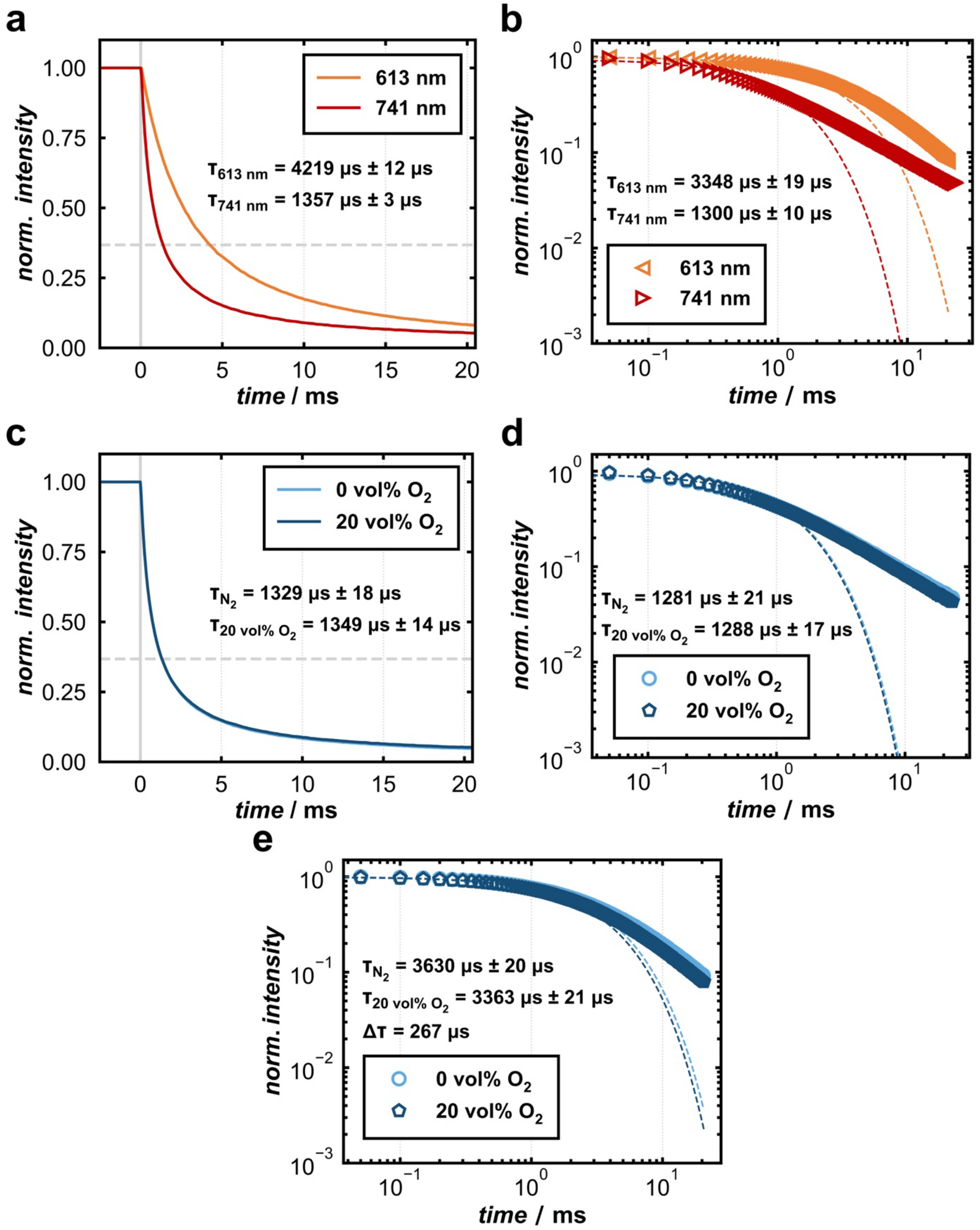

Figure S5. ***a*** *lifetimes at 613 nm and 741 nm (ambient conditions), τ is (1/e),* ***b*** *lifetimes at 613 nm and 741 nm (ambient conditions), τ from single exponential fit,* ***c*** *lifetimes at 741 nm in pure N_2 and 20 vol% O_2 in N_2 atmosphere, τ is (1/e),* ***d*** *lifetimes at 741 nm in pure N_2 and 20 vol% O_2 in N_2 atmosphere, τ from single exponential fit,* ***e*** *lifetimes*

at 613 nm in pure N_2 and 20 vol% O_2 in N_2 atmosphere, τ from single exponential fit. All excited with 325 nm.

Measurement Setup

On a ceramic substrate with a gold interdigital structure the conductance and photoluminescence (PL) of the drop-coated ZTO particles can be measured simultaneously with a custom-built setup. The measurement setup (figure S6a) is housed in an aluminum body to shield external light. The insert for a commercially available quartz glass cuvette (figure S6b) was constructed to measure PL and conductance at a controlled temperature and gas atmosphere. A type-K thermocouple is connected to a flue gas analyzer (Woehler A450) to determine the substrate temperature. An Agilent E3640A power supply was used for heating; the heating element is controlled by a Python script. A Roithner DUV325-HL46N LED, operated at 15 V with a Voltcraft LSP-1403 power supply, was used to excite the sample. The emitted radiation is collected with a collimator lens and transferred by optical fibers to an Ocean Optics Flame Miniature spectrometer. The gas atmosphere is mixed with a gas mixing system (Woehler GM450) and applied with a flow rate of 150 mL/min. The interdigital electrode structure is operated in series with a 1 MOhm resistor on a Voltcraft LSP-1403 power supply with a constant output voltage of 20 V. The conductance of the ZTO particles is calculated using the measured voltage (multimeter, HP 34401A) via the interdigital structure.

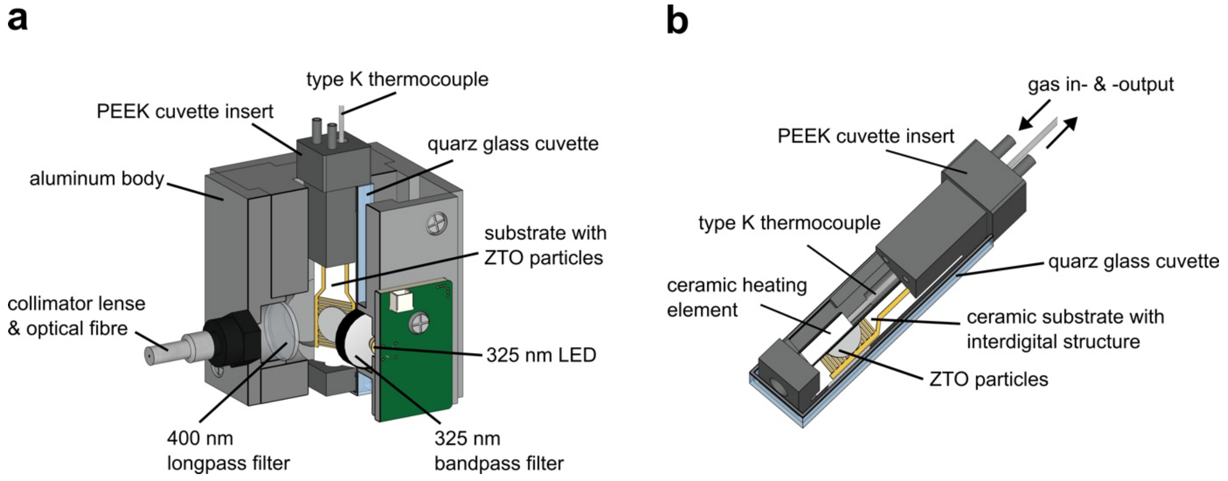

Figure S6. *Sectional drawings of* ***a*** *experimental setup to measure photoluminescence and conductance simultaneously at controlled temperatures and gas atmospheres,* ***b*** *the cuvette insert.*

Data Evaluation

For each emission spectrum the spectrometer integrates over 30 seconds. To evaluate the data, spectra are smoothed with a moving average of 20 measurement points. The spectral resolution is 0.473 nm. The emission spectrum of ZTO particles is displayed in figure S7. To evaluate the quencher impact, the intensity at 606 nm (vertical orange line) is determined for every spectrum and tracked over time.

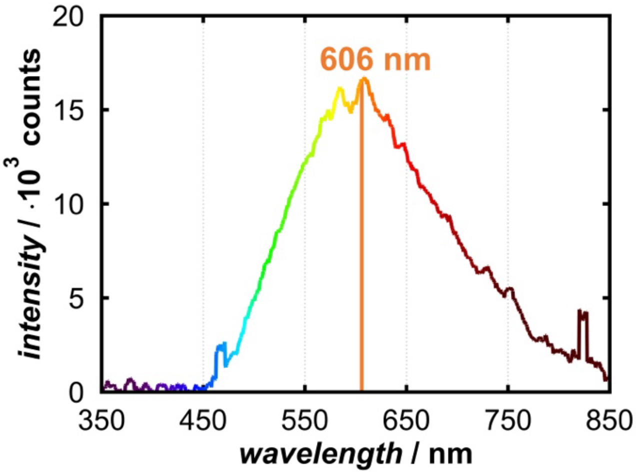

Figure S7. *emission spectrum of ZTO particles, 606 nm highlighted with vertical orange line, excited with 325 nm.*

Measurement Modes

Two different measurement modes were performed. During the first one, the O_2 concentration was kept constant during the measurement, and the temperature was varied. Then the temperature was kept constant during the measurement, and the O_2 concentration varied. For the first measurement mode (figure S8a), the temperature is varied in 0.5 vol%, 1 vol%, 2.5 vol%, 5 vol%, 10 vol%, 15 vol% and 20 vol% O_2 in N_2 atmospheres (150 ml/min). The temperature sequence starts with a 150 °C segment for 2 h, followed by a temperature decrease to 40 °C in 10 °C steps. Each temperature is kept constant for 30 min. Finally, an additional segment at 150 °C is applied for 15 min. The second measurement mode (figure S8b) is operated at 40 °C, 80 °C, 120 °C and 160 °C. After a 4 h N_2 segment the O_2 concentration is varied from 0.5 vol% to 20 vol% for 1 h each (150 ml/min). The measurements performed to determine the lower detection limit were performed at 120 °C. After a 4 h N_2 segment the O_2 concentration varied from 0.33 vol% to 0.7 vol% for 1 h each (300 ml/min).

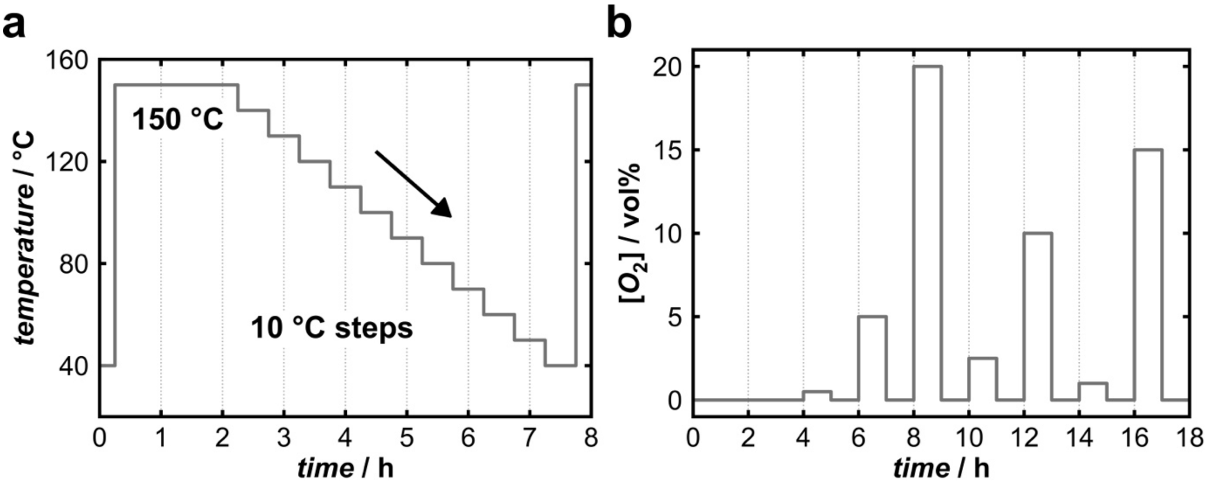

Figure S8. ***a*** *temperature profile under constant O_2 concentration,* ***b*** *concentration profile at constant temperatures*

Temperature variation (0% to 20 vol% O_2 in N_2)

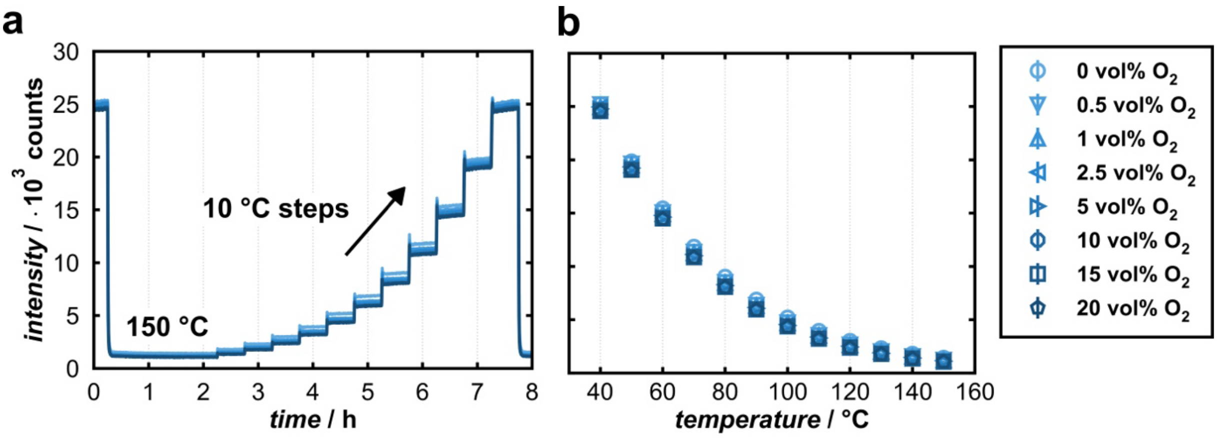

Figure S9. ***a*** *temperature profile under constant O_2 concentration,* ***b*** *concentration profile at constant temperatures.*

Derivation of Modified Stern-Volmer Equation

$$f_a = \frac{I_{0,a}}{I_0} \rightarrow I_{0,a} = f_a\, I_0 \tag{S2}$$

$$I_0 = I_{0,a} + I_{0,b} \quad \rightarrow \quad I_{0,b} = I_0 - f_a\, I_0 \tag{S3}$$

$$I_Q = \frac{I_{0,a}}{1 + K_a[O_2]} + I_{0,b} \tag{S4}$$

$$I_Q = \frac{f_a I_0}{1 + K_a[O_2]} + I_0 - f_a\, I_0 \tag{S5}$$

$$I_Q = I_0 \left(\frac{f_a}{1 + K_a[O_2]} + 1 - f_a \right) \qquad | : I_0 \tag{S6}$$

$$\frac{I_Q}{I_0} = \frac{f_a}{1 + K_a[O_2]} + 1 - f_a \qquad | - 1 \tag{S7}$$

$$\frac{I_Q}{I_0} - 1 = \frac{f_a}{1 + K_a[O_2]} - f_a \qquad | \cdot (-1) \tag{S8}$$

$$1 - \frac{I_Q}{I_0} = f_a - \frac{f_a}{1 + K_a[O_2]} \tag{S9}$$

$$\frac{I_0}{I_0} - \frac{I_Q}{I_0} = f_a \left(1 - \frac{1}{1 + K_a[Q]} \right) \qquad | : f_a \tag{S10}$$

$$\frac{I_0 - I_Q}{I_0 f_a} = \frac{1 + K_a[Q]}{1 + K_a[Q]} - \frac{1}{1 + K_a[Q]} = \frac{1 + K_a[Q] - 1}{1 + K_a[Q]} = \frac{K_a[Q]}{1 + K_a[Q]} \qquad | \cdot f_a \tag{S11}$$

$$\frac{I_0 - I_Q}{I_0} = \frac{f_a K_a[Q]}{1 + K_a[Q]} \qquad | \; (\;)^{-1} \tag{S12}$$

$$\frac{I_0}{I_0 - I_Q} = \frac{1 + K_a[Q]}{f_a K_a[Q]} = \frac{1}{f_a K_a[Q]} + \frac{K_a[Q]}{f_a K_a[Q]} = \frac{1}{f_a K_a[Q]} + \frac{1}{f_a} \tag{S13}$$

Conductance & Photoluminescence Measurements

The conductance was normalized on the maximum value at each temperature, table S1.

Table S1. Maximum conductance between 40 °C and 160 °C.

temperature / °C	conductance / nS
40	352.87
80	426.98
120	596.00
160	1046.42

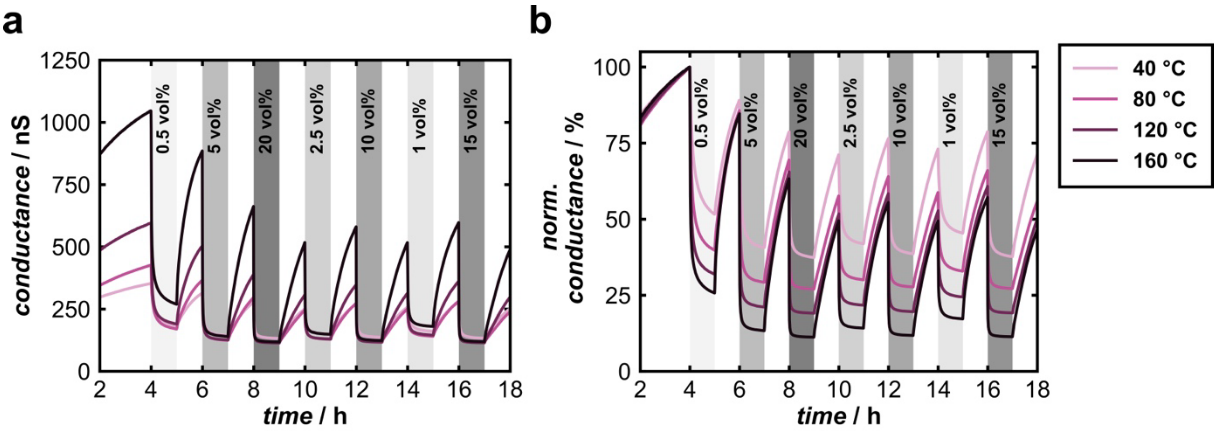

Figure S10. *O_2 dependent conductance between 40 °C and 160 °C* ***a*** *as measured,* ***b*** *normalized.*

The intensity was averaged between 3 h and 4 h and used for normalization at each temperature, table S2.

Table S2. Maximum intensity between 40 °C and 160 °C.

temperature / °C	intensity / counts
40	33039
80	16493
120	6586
160	2541

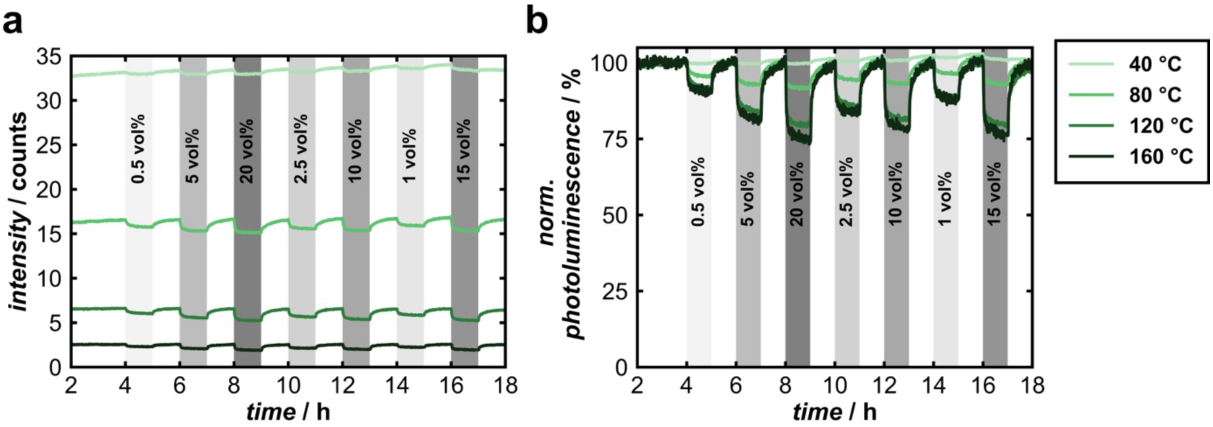

Figure S11. *O_2 dependent photoluminescence between 40 °C and 160 °C* ***a*** *as measured,* ***b*** *normalized.*

From the measurements displayed in Figure 6 the characteristic curves at the different operating temperatures were extracted for conductance and photoluminescence.

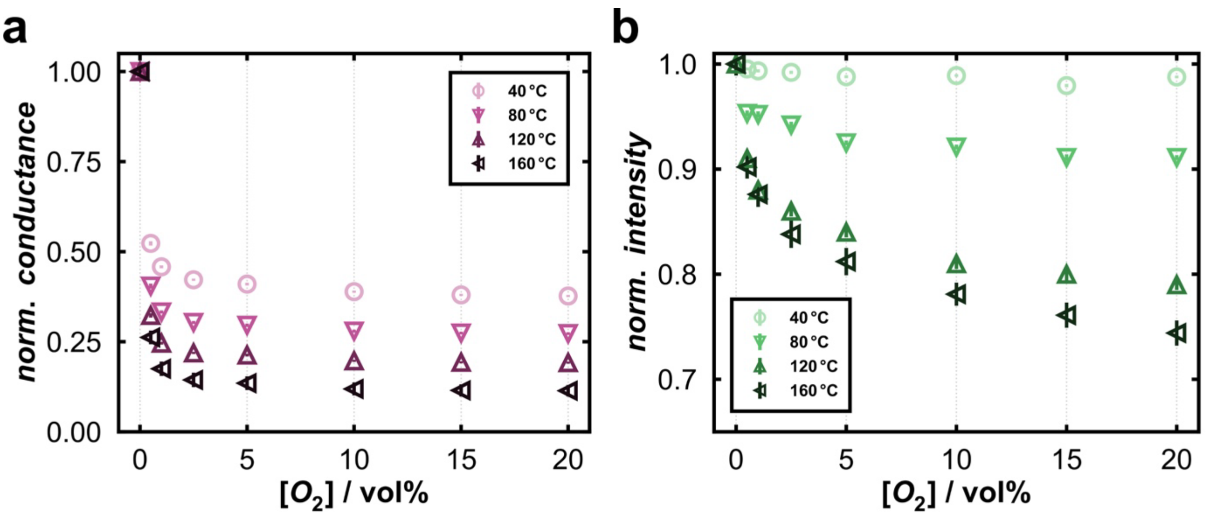

Figure S12. *O_2 dependent responses for* ***a*** *conductance and* ***b*** *photoluminescence between 40 °C and 160 °C.*

Table S3. Maximum conductance at 160 °C with and without optical activation.

optical activation	conductance / nS
325 nm	1046.42
without	140.19

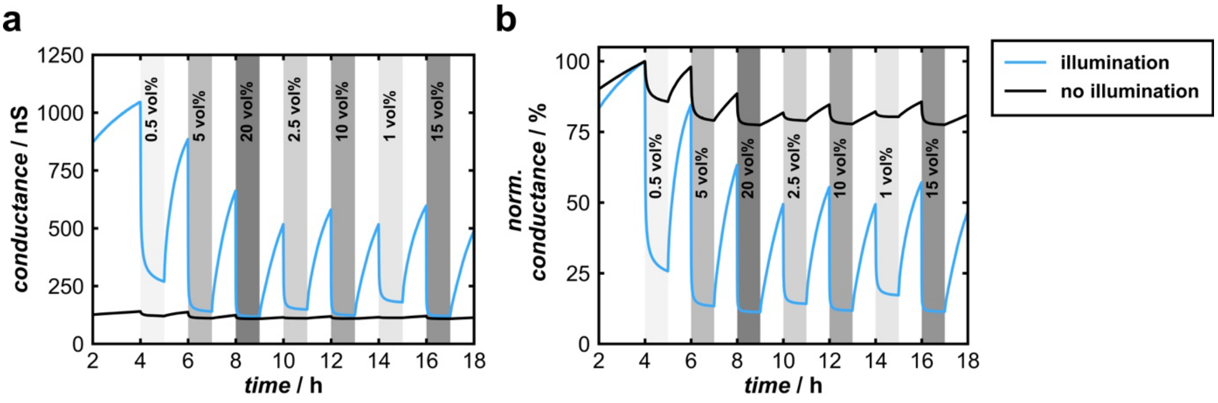

Figure S13. *O_2 dependent conductance at 160 °C with illumination by 325 nm LED (light blue) and without illumination (black)* ***a*** *as measured,* ***b*** *normalized.*

Determination of lower detection limit from simultaneous measurement of photoluminescence and conductance. Lower O_2 concentrations could not be measured due to the current gas mixing equipment.

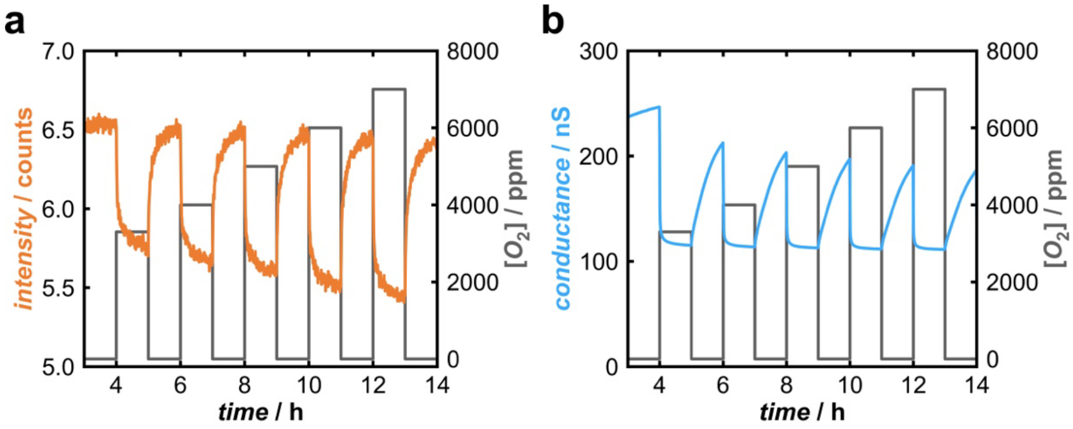

Figure S14. *photoluminescence* ***a*** *and conductance* ***b*** *measured simultaneously at 120 °C at different* O_2 *concentrations (3300 ppm to 7000 ppm).*

Table S4. Maximum intensity and conductance at 120 °C.

intensity	6530 counts
conductance	245.84 nS
